# Two Functionally Distinctive Phosphopantetheinyl Transferases from Amoeba *Dictyostelium discoideum*

**DOI:** 10.1371/journal.pone.0024262

**Published:** 2011-09-12

**Authors:** Divya R. Nair, Ratna Ghosh, Alzu Manocha, Debasisa Mohanty, Shweta Saran, Rajesh S. Gokhale

**Affiliations:** 1 National Institute of Immunology, New Delhi, India; 2 School of Life Sciences, Jawaharlal Nehru University, New Delhi, India; 3 Institute of Genomics and Integrative Biology, Council of Scientific and Industrial Research, Delhi, India; 4 Jawaharlal Nehru Centre for Advanced Scientific Research, Bangalore, India; University of South Florida College of Medicine, United States of America

## Abstract

The life cycle of *Dictyostelium discoideum* is proposed to be regulated by expression of small metabolites. Genome sequencing studies have revealed a remarkable array of genes homologous to polyketide synthases (PKSs) that are known to synthesize secondary metabolites in bacteria and fungi. A crucial step in functional activation of PKSs involves their post-translational modification catalyzed by phosphopantetheinyl transferases (PPTases). PPTases have been recently characterized from several bacteria; however, their relevance in complex life cycle of protozoa remains largely unexplored. Here we have identified and characterized two phosphopantetheinyl transferases from *D. discoideum* that exhibit distinct functional specificity. DiAcpS specifically modifies a stand-alone acyl carrier protein (ACP) that possesses a mitochondrial import signal. DiSfp in contrast is specific to Type I multifunctional PKS/fatty acid synthase proteins and cannot modify the stand-alone ACP. The mRNA of two PPTases can be detected during the vegetative as well as starvation–induced developmental pathway and the disruption of either of these genes results in non-viable amoebae. Our studies show that both PPTases play an important role in *Dictyostelium* biology and provide insight into the importance of PPTases in lower eukaryotes.

## Introduction

*Dictyostelium discoideum* (Dicty) is a unicellular amoeba that undergoes multicellular differentiation when faced with starvation. This development process results in the formation of a fruiting body, which contains viable spores that germinate on return of favourable conditions. Various small metabolites are known to play a crucial role during this morphogenesis [1]–[6]. Two of the important developmental regulating factors (DRFs) – Differentiation Inducing Factor (DIF) and 4-methyl-5-pentylbenzene-1,3-diol (MPBD) are synthesized by large multifunctional polyketide synthases (PKSs) [7], [8]. The genome of this organism has revealed an astoundingly large number of Type I PKSs [9], which are known to utilize a thio-template-based mechanism of biosynthesis. In this mechanism both starter and intermediate moieties are covalently acylated as thioesters either on the cysteine residue of ketosynthase (KS) domain or on the phosphopantetheine arm of acyl carrier protein (ACP). Phosphopantetheinyl modification of the ACP domains is a post-translational event and is essential for activity of PKSs. This modification is catalyzed by a group of enzymes known as phosphopantetheinyl transferases (PPTases) [10], [11]. Several studies in recent years have revealed interesting insights into these enzymes; however, significance of PPTases in developmentally complex organisms is still obscure [12]–[22].

**Figure 1:**
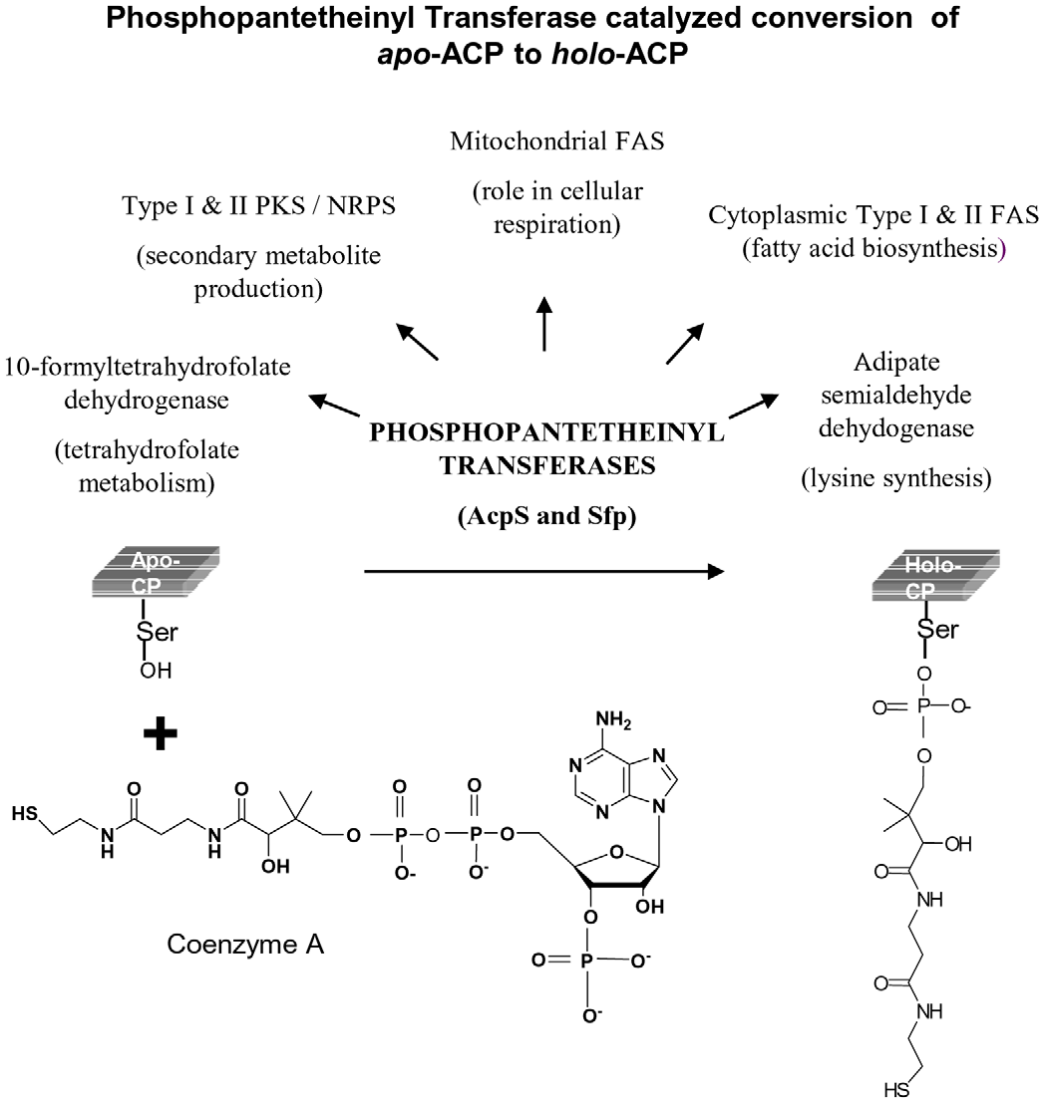
Schematic representation of phosphopantetheinyl transferase (PPTase) catalyzed conversion of *apo*-ACP to *holo*-ACP. Phosphopantetheine group of coenzyme A is transferred by the PPTases to a conserved serine group of ACP. This post-translational modification of carrier proteins is necessary for their function in various metabolic pathways.

PKSs consist of three core catalytic domains – acyltransferase (AT), ACP and KS that act in a concerted manner with auxilliary domains to yield the final product. In Type I PKSs various domains are present on a single polypeptide chain. In Type II PKSs these domains are present as discrete proteins that form a non-covalent complex. Type III PKSs are simple homodimeric proteins, and until recently were not known to require any carrier domains during repetitive condensations. In recent years, carrier domains have also been shown to provide starter units for Type III PKSs [7], [8]. The modification of carrier domains involves the transfer of a 340 Da phosphopantetheine group derived from coenzyme A (CoA) on to the hydroxyl group of serine residue of ACP domains (Fig. 1). PPTases are also required for activating carrier proteins (CPs) of fatty acid synthases (FASs), non-ribosomal peptide synthases (NRPSs), adipate semialdehyde dehydrogenase [23] and 10′-formyl tetrahydrofolate dehydrogenase [24].

Conventionally, PPTases have been broadly categorized into two families [12]. The prototype for the first family of enzymes is the Sfp protein from *Bacillus subtilis*. Sfp-like PPTases are approximately 34 kDa enzymes. These proteins exhibit promiscuous capability to post-translationally modify wide range of carrier proteins. The members of second family of enzymes, designated as AcpS, are relatively small (about half the size) and are known to be generally more stringent in choosing carrier proteins. This family also includes a separate subfamily of integrated PPTases involved in fungal FAS [11]. Interestingly, both Sfp and AcpS can utilize acyl-CoAs as substrates and directly load the acyl-phosphopantetheine group on to the carrier proteins [25]–[27].

Functions of PPTases in different organisms have revealed interesting themes. The genome of *B. subtilis* encodes for AcpS and Sfp, former is involved in fatty acid synthesis and the latter in surfactin production [12]. However, in the absence of AcpS, Sfp has been shown to take over its function. This is in contrast to *Mycobacterium tuberculosis* where the roles of AcpS and Sfp have been reported to be non-redundant in nature [16]. The three PPTases from *Myxococcus xanthus* are categorized into - AcpS that is involved in fatty acid biosynthesis and two redundant Sfp proteins, modifying enzymes of secondary metabolic pathway [13]. In certain examples PPTases are known to be associated specifically with enzyme systems [18]. In parasites, the function of two PPTases is suggested to be dictated by the architecture of the proteins [17]. In *Toxoplasma gondii*, the two PPTases have been proposed to cater to the Type I and Type II FAS systems. Interestingly, in *Cryptosporidium parvum* a single PPTase, Sfp is capable of modifying multifunctional Type I PKS and FAS and it does not contain AcpS. *Plasmodium*, which possesses only Type II FAS, has AcpS for mediating activation of CP. In fungi such as *Aspergillus nidulans* one of the three PPTases is an integral component of the FAS α-subunits and another one is suggested to be involved in activation of a mitochondrial FAS [14]. The third PPTase is demonstrated to be Sfp-like protein that is responsible for the activation of all its 27 PKSs and 14 NRPSs.

These studies clearly highlight that despite characterization of several different Sfp and AcpS-like PPTases, even now it is not possible to predict their selectivity for different protein types. It is therefore important to characterize them and delineate their functional specificities in a case-specific manner.

In this study, we have investigated the functional role of two PPTase homologues in *Dictyostelium* biology. Here, we demonstrate that the two PPTases, DiAcpS and DiSfp are functionally discrete and non-redundant in nature. Furthermore, biochemical studies unambiguously show that DiSfp is required for the activation of multifunctional PKS/FAS, whereas, DiAcpS can modify only the stand-alone ACP. We also show that both PPTases are expressed during all the stages of its life cycle. Through our studies we demonstrate the importance of PPTases in the life cycle of *Dictyostelium*.

## Results and Discussion

### Identification of Dictyostelium PPTases

**Figure 2:**
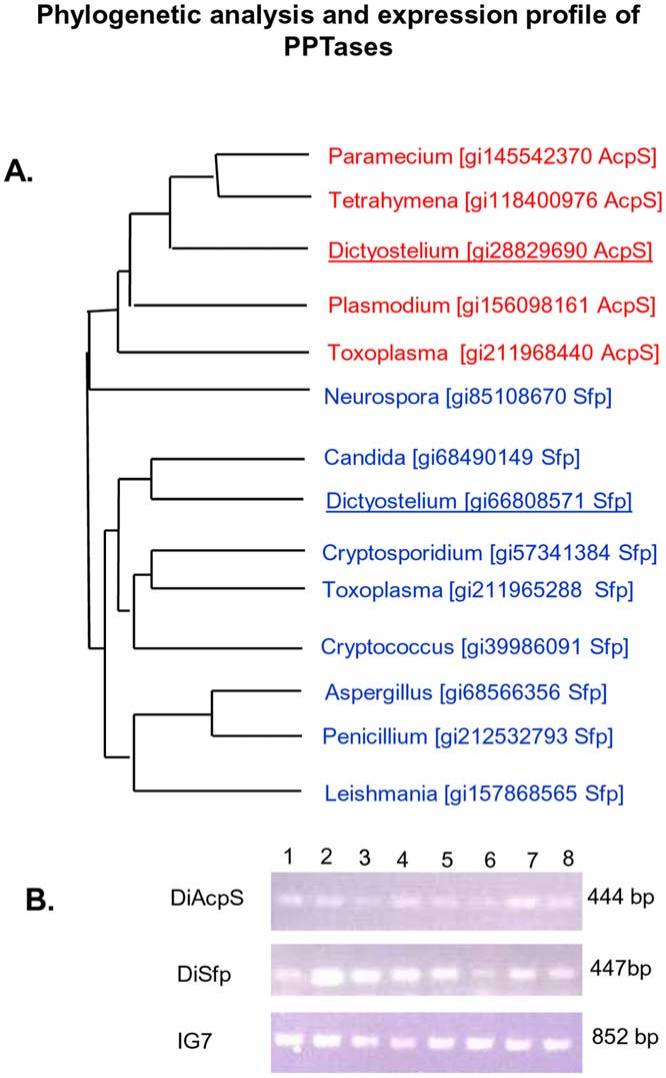
Phylogenetic analysis and expression profile of PPTases. A, Sequences of PPTases from *Dictyostelium* and other lower eukaryotes were analyzed for their evolutionary relatedness. The sequences branch into two distinct groups, one group constituting AcpS-like sequences and the other formed by Sfp-like sequences. B, Expression profiles of DiAcpS and DiSfp were studied by RT-PCR and both were found to be expressing at all developmental stages. Stages analyzed were amoeboid (1), 0 hrs. after starvation (2), streaming (3), loose aggregate (4), mound (5), slug (6), early culminant (7) and fruiting body (8). IG7 (mitochondrial large rRNA) was used as the RT-PCR control.

The presence of 45 PKSs in *Dictyostelium* is quite unprecedented and to understand their relevance in multicellular development, we first decided to investigate into the modes by which PKSs are post-translationally modified by PPTases. We performed BLAST searches with *Streptococcus pneumoniae* AcpS and *B. subtilis* Sfp on Dictybase (www.dictybase.org) to identify PPTase-like sequence(s) in the Dicty genome. Our analysis revealed the presence of two protein sequences with considerable homology. DDB0217726 (DiAcpS) shows 45% similarity with *S. pneumoniae* AcpS, whereas, DDB0186752 (DiSfp) exhibits 53% similarity with *B. subtilis* Sfp. Both these proteins show motifs characteristic of PPTases - [IV]G[ITV]D[ILV][VE] and W[CA][AL]KEAxxK. In addition, DiSfp also contains the (FNxSH) motif that is characteristic of Sfp type PPTases [17]. Comparative analysis of these protein sequences based on the crystal structures of *B. subtilis* AcpS and Sfp [28], [29] also showed conservation of key residues. This includes R16 and R23 residues in DiAcpS involved in recognition of ACP and so also the amino acids D8, F25, R28, E58 and F74 (numbering corresponds to *Bacillus* AcpS) reported to be involved in binding of CoA and Mg^2+^. Careful analysis of DiSfp sequence also revealed the presence of residues – H118, S117, K52 and K193 which have been shown in *B. subtilis* Sfp to be involved in CoA binding. Dendrogram-based phylogenetic analyses of Dicty PPTase sequences with other lower eukaryotes, including fungi and protozoan parasites readily provided their classification in two distinct groups of AcpS-like and Sfp-like sequences (Fig. 2A). Putative Sfp homologue from *Neurospora* does not group itself in either of the two clades. In order to understand the relevance of the two putative PPTases from Dicty, we decided to carry out genetic and biochemical investigations.

### Gene knockout and expression studies of PPTases

In order to generate genetic-knockouts of the individual genes, we adopted standard homologous recombination techniques. After transformation of the linear cassette (Figure S1), the Dicty amoeboid cells were aliquoted into 96×4 wells. On systematic increase of antibiotic selection, ∼50% wells showed growth of Dicty cells in the case of *diacps*. For *disfp* knockout transformants, survival rate was ∼20%. PCR-based analysis was performed on 124 *diacps* clones and 96 *disfp* clones to confirm homologous recombination. However, in all the cases clones were found to be non-homologous recombinants (Figure S2). In order to validate experimental strategy, *dipks37* knockout was also attempted. Five positive clones for *dipks37* knockout could be confirmed and these mutants showed similar phenotypic characteristics as described earlier in the literature [7] (Figure S3). We attempted few different primer sets for PPTases, however, all our attempts provided only non-homologous recombinants. Our genetic studies could not provide any further information and therefore we proceeded to examine the presence of gene transcripts through different stages of this organism. RT-PCR analysis indicated presence of mRNA levels of both PPTases during all the stages of the life cycle of Dicty (Fig. 2B). Since the genetic tools for making conditional mutants or for RNAi studies are not robust in Dicty, we decided to biochemically delineate the functional implications of these proteins.

### Cloning and expression of Phosphopantetheinyl Transferases

Both the proteins were heterologously expressed in *E. coli. Diacps* gene has 2 exons, which were amplified using nested primers from genomic DNA. Similarly *disfp* also has 2 exons and these were PCR amplified individually and cloned into expression vector. Proteins were expressed by using a T7-based expression system and both the proteins could be readily purified using affinity chromatography. DiAcpS showed a band at around 27 kDa on SDS-PAGE rather than the expected molecular mass of 20 kDa. Meanwhile, DiSfp showed the theoretical molecular mass of 34 kDa. Identities of both the proteins were confirmed using mass spectrometric analysis (Figure S4).

### Cell-free Assay with Mycobacterial Type I PKS

**Figure 3:**
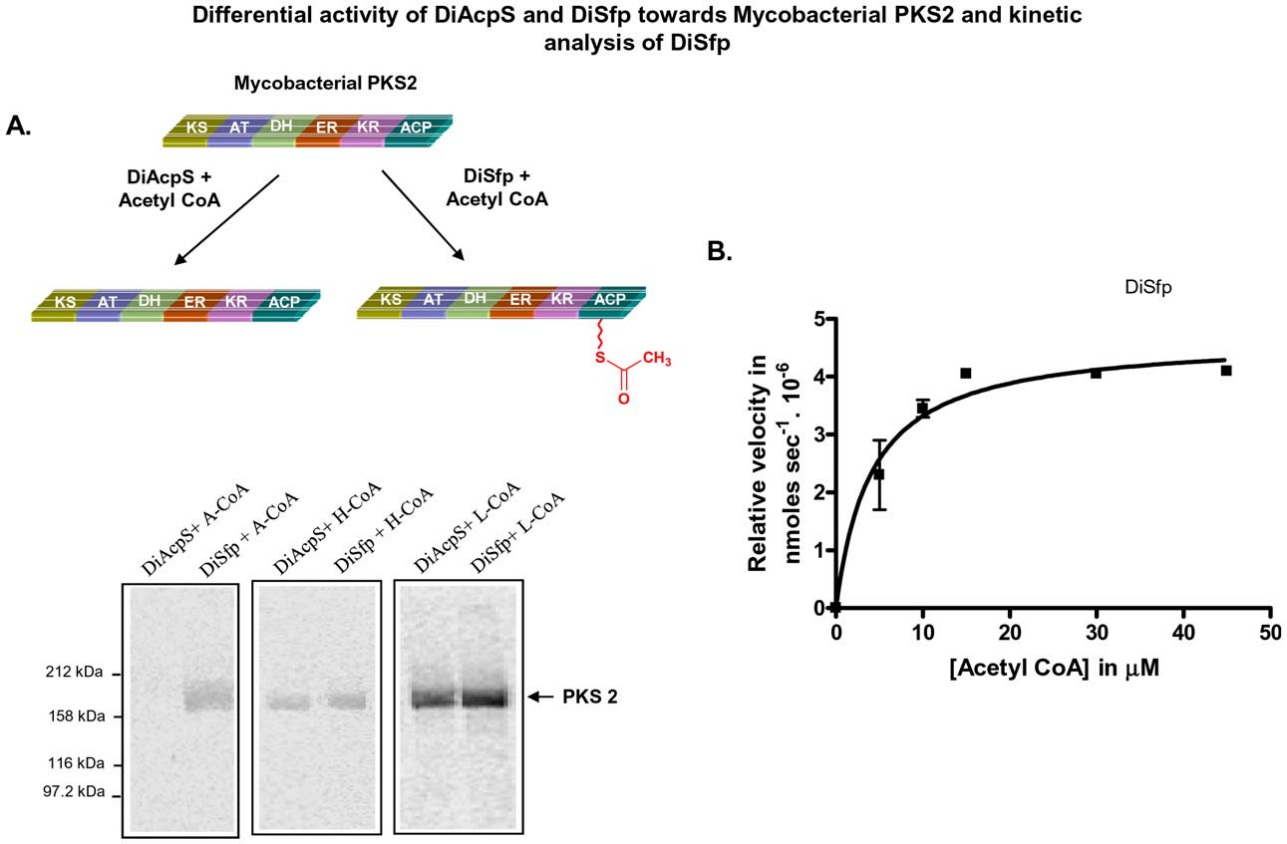
Differential specificity of *Dictyostelium* Sfp (DiSfp) towards mycobacterial PKS2 and kinetic analysis. Gel-binding assays were set up with DiAcpS and DiSfp, taking [1-^14^C] acetyl-CoA (A-CoA), [1-^14^C] hexanoyl-CoA (H-CoA) and [1-^14^C] lauroyl-CoA (L-CoA) as co-substrates. A, Panels show the autoradiogram of SDS-PAGE in which mycobacterial PKS2 was labeled by different acyl CoA substrates and PPTases as indicated. B, Gel-based kinetic analysis of DiSfp with respect to acetyl CoA. Concentration of acetyl CoA was varied from 5 µM to 45 µM.

Preliminary analysis of the enzymatic activity of the PPTases was performed with multifunctional Type I mycobacterial PKSs. Several of these PKSs have been previously cloned and functionally characterized in our laboratory. Radiolabeled [1-^3^H] CoA was used as substrate and the transfer of radiolabel was detected by autoradiography. Whereas weak signal could be obtained with DiSfp, DiAcpS did not show any radioactive band. The profile remained unaltered even after increasing the concentration of DiAcpS and/or long exposure times. Since PPTases are also known to use acyl CoA as substrates [26], [27], [30], we used [1-^14^C] acetyl CoA for our further studies. Assays set up with PKS2, PKS12 and MAS (mycocersoic acid synthase) along with labeled acetyl CoA clearly indicated phosphopantetheinylation only by DiSfp and no activity could be observed for DiAcpS (Fig. 3A). Catalytic efficiency of phosphopantetheine transfer with respect to acetyl CoA was estimated by Michaelis-Menten fit and k_cat_/K_m_ was calculated to be 0.353 mM^−1^ min^−1^ (Fig. 3B). The ACP concentration was fixed to be approximately 2.5-fold higher than the PPTase enzymes. We further probed if DiSfp was capable of transferring longer chain acyl-CoAs (hexanoyl and lauroyl CoA) on PKSs. Surprisingly, not only DiSfp but also DiAcpS showed ability to transfer both hexanoyl CoA and lauroyl CoA with comparable efficiencies (Fig. 3A). This unusual ability to only transfer longer chain acyl-CoA by DiAcpS on type I PKS was perplexing.

### Comparative analysis of PPTase activity with type II stand-alone ACP

**Figure 4:**
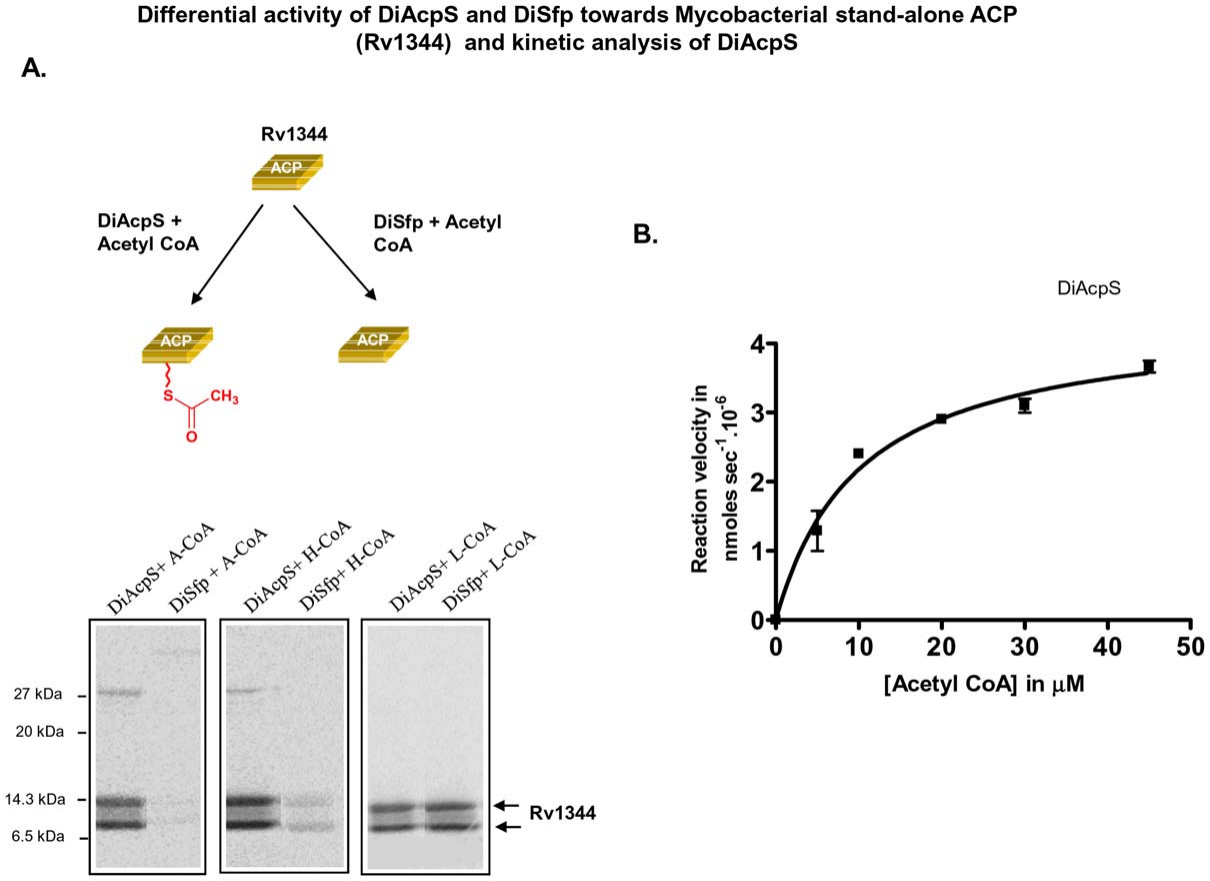
Differential specificity of *Dictyostelium* AcpS (DiAcpS) towards mycobacterial stand-alone ACP and kinetic analysis. A, Panels show the autoradiogram of SDS-PAGE in which mycobacterial Rv1344 was labeled by different acyl CoA substrates and PPTases as indicated. Rv1344 shows the presence of two protein bands, lower one being the N-terminus truncated form. The truncated form is also seen to be incorporating radioactivity, suggestive of PPTase mediated modification. B, Michaelis-Menten kinetic analysis of DiAcpS was performed in a similar way as that for DiSfp.

**Figure 5:**
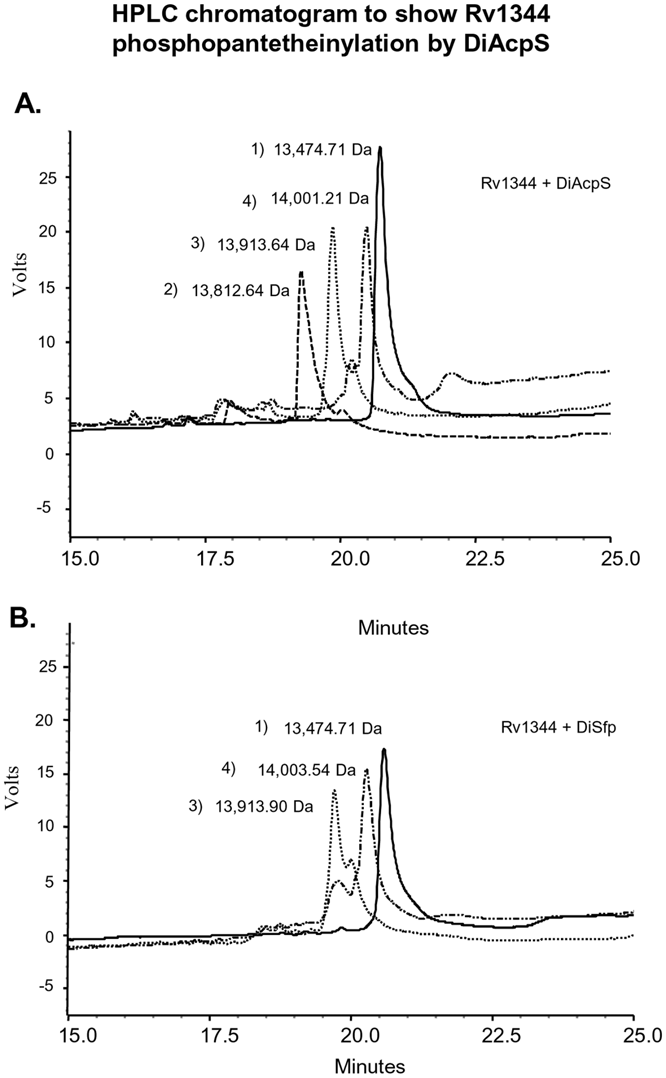
Rv1344 phosphopantetheinylation by DiAcpS. A, HPLC chromatogram of assays with DiAcpS. B, HPLC chromatogram for DiSfp reactions. Reactions were carried out with coenzyme A (peak 2), hexanoyl CoA (peak 3) and lauroyl CoA (peak 4). Peak 1 represents *apo*-form of the ACP in each case. Each peak was subjected to MALDI-TOF analysis and the molecular masses obtained for each peak have been indicated.

To ascertain the activity of PPTases with type II ACP, mycobacterial stand-alone ACP (Rv1344) was chosen. The purified protein shows two bands on SDS-PAGE, a 13 kDa band corresponding to the intact protein size and an 11 kDa band, which possesses truncation at the N-terminus. Gel binding assays with this stand-alone carrier protein showed a reverse trend for the function of two PPTases in comparison to studies with Type I PKSs (described above). While DiAcpS could efficiently catalyze phosphopantetheinylation of Rv1344, DiSfp exhibited weak activity with acetyl-CoA (Fig. 4A). Kinetics of DiAcpS with the ACP was determined by varying the concentrations of acetyl CoA and the k_cat_/K_m_ was estimated to be 0.162 mM^−1^ min^−1^ (Fig. 4B). In order to unambiguously confirm phosphopantetheinylation of ACP, these proteins were separated on reverse-phase column and subjected to mass spectrometric analysis. DiAcpS mediated conversion of *apo*- to *holo*-ACP resulted in shift in the retention time of the protein from 20.7 minutes to 19.4 minutes (Fig. 5A). The peak at 19.4 minutes showed an increment of 340 Da, confirming modification with phosphopantetheine group. The enzymatic assays of *apo*-ACP (13,474 Da) with hexanoyl CoA and lauroyl CoA exhibited higher molecular masses of 13,913 Da and 14,003 Da respectively, consistent with attachment of corresponding acyl-phosphopantetheine group. DiSfp could not efficiently catalyze conversion of *apo*-type II ACP to the *holo*-form, but showed reasonable activity with hexanoyl and lauroyl-CoAs (Fig. 5B).

### Analysis of DiSfp and DiAcpS with ACP fragment of Mycobacterial PKS12

**Figure 6:**
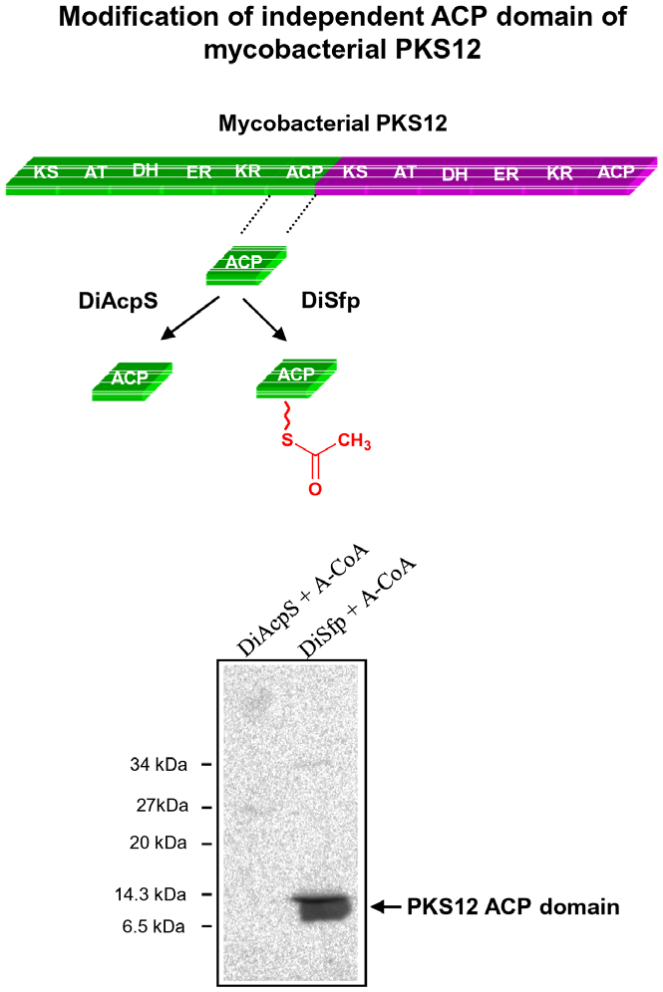
Modification of independent ACP domain of mycobacterial PKS12. Autoradiography shows acetyl-phosphopantetheinylation of the independent ACP domain of multi-functional PKS by DiSfp. DiAcpS is unable to incorporate radioactivity into the ACP.

The specificity of DiAcpS and DiSfp to phosphopantetheinylate type II ACP and type I PKS ACP respectively prompted us to examine whether this selectivity is due to architectural differences of the PKS proteins or is an inherent property of ACP domain. To resolve this issue, we cloned and expressed the PKS12 (Type I PKS) ACP fragment and then studied its phosphopantetheinylation by the two PPTases. Boundaries of the module 1 ACP were defined using the PKS-NRPS database [31]. Gel-binding assay with the purified protein showed that the transfer of phosphopantetheine group on to the isolated PKS12 ACP domain could be catalyzed solely by DiSfp. This is in concordance with the data wherein this ACP as part of the larger polypeptide could not be modified with DiAcpS (Fig. 6). Clearly, our work shows that the specificity of phosphopantetheinylation is not dictated by the modular architecture, but in fact involves specific recognition of the carrier domains. An earlier study with rat FAS ACP domain however, had come to reverse conclusion [32]. In that case an independently expressed ACP domain from multifunctional FAS system could be modified by bacterial AcpS that is known to modify Type II FAS ACP proteins. This apparent contradiction could be a manifestation of a broader specificity of AcpS protein or could also be argued in terms of the projected evolutionary relationship of two PPTases with primary and secondary metabolism [12], [16], [23], [26].

### Studies on Dictyostelium Type I PKS/FAS

**Figure 7:**
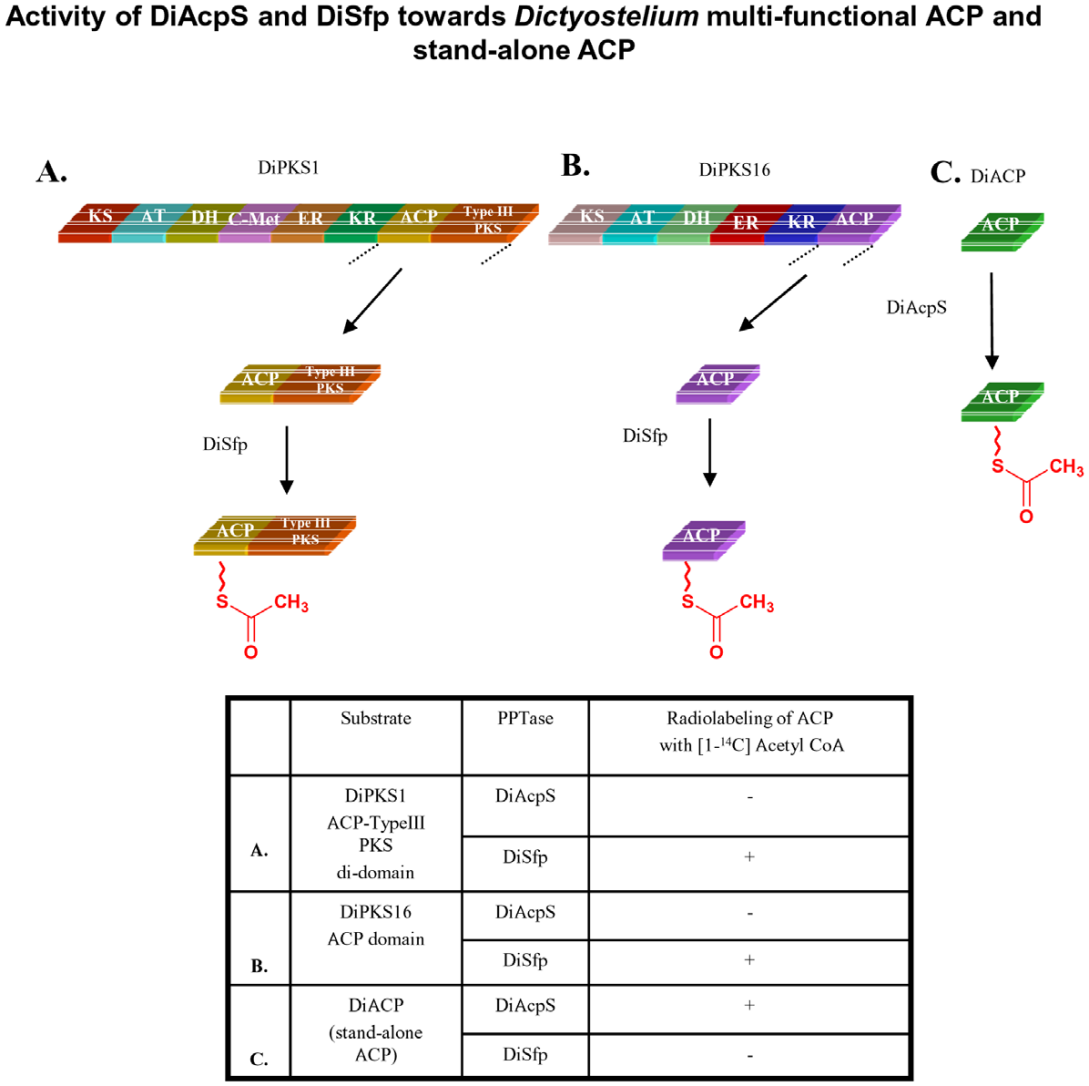
Activity of DiAcpS and DiSfp towards *Dictyostelium* multi-functional ACP and stand-alone ACP. Gel-binding assays were performed with radioactive acetyl CoA (A-CoA) to confirm the specificities of the PPTases with cognate ACPs. A, ACP-TypeIII PKS di-domain of DiPKS1 shows radiolabeling with DiSfp, but not with DiAcpS. B, ACP domain of DiPKS16 is also modified just by DiSfp. C, the stand-alone ACP, DDB0184099, shows converse pattern. DiAcpS is able to mediate the conversion of *apo*-form of this ACP to *holo*-form and DiSfp fails to show activity.

The activity of Dicty PPTases was then evaluated by using cognate proteins from the organism. Since experiments with mycobacterial proteins suggested that modular arrangement does not determine the selectivity, we used a smaller di-domain protein to understand phosphopantetheinylation specificity. ACP-TypeIII PKS di-domain of DiPKS1 (DDB0234164) was used for the assays with both PPTases [8]. Figure 7A illustrates that DiSfp is able to phosphopantetheinylate ACP-TypeIII PKS but DiAcpS fails to convert this *apo*-protein to its *holo* form. This reinforces our view that DiAcpS cannot function with type I PKS.

**Figure 8:**
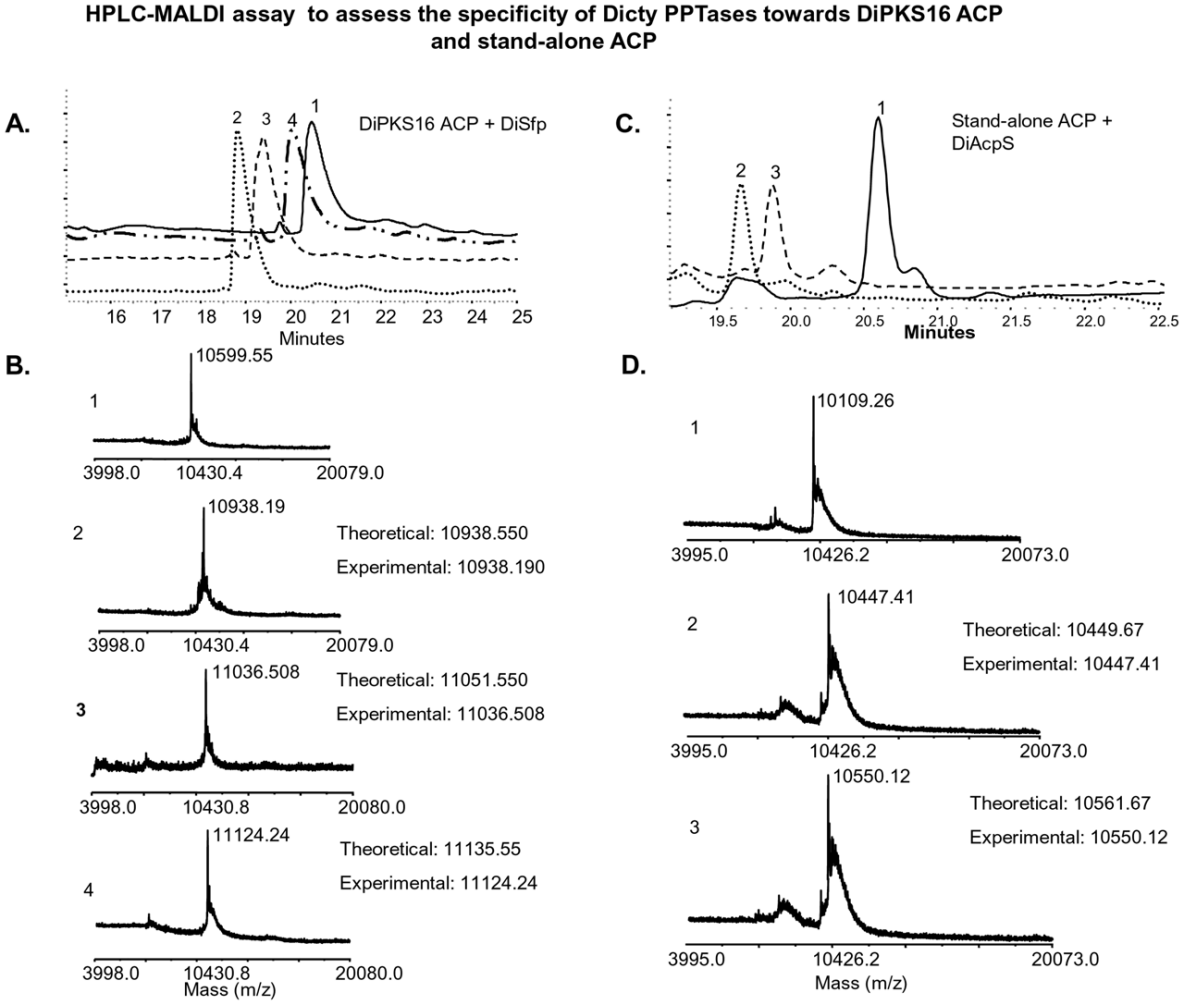
HPLC-MALDI assays for assessing DiSfp and DiAcpS activity towards DiPKS16 ACP domain and stand-alone ACP respectively. A, HPLC chromatogram of DiSfp reactions with DiPKS16 ACP. Reactions with coenzymeA, hexanoyl CoA and lauroyl CoA are illustrated as peaks 2, 3 and 4 respectively. Peak 1 in each case corresponds to *apo*-ACP. B, MALDI-TOF analysis of the peaks obtained in Fig. 8A is depicted. C, HPLC traces of DiAcpS reactions with *Dictyostelium* stand-alone ACP are represented. D, MALDI-TOF analysis of the peaks obtained in Fig. 8C is shown.

AcpS in several studies is suggested to be involved in primary metabolism of fatty acids [12], [16], [23], [26]. We therefore wanted to investigate if similar scenario exists in Dicty. DiPKS16 (DDB0230068) was chosen as the candidate protein to test this hypothesis because this protein has been implicated as putative type I FAS [33]. DiPKS16 ACP domain boundaries were determined using the PKS-NRPS database [31]. The 258 bp gene was induced to over-express the C-terminus His_6_ tagged protein in *E. coli*. The purified ACP showed anomalous mobility on SDS-PAGE, indicating a molecular mass of ∼27 kDa rather than the calculated mass of 10.6 kDa. Similar anomalous migration of ACPs has been reported in literature and is attributed to their highly acidic nature [34]. Mass spectrometric analysis established the protein to be DiPKS16 ACP (Figure S5) Phosphopantetheinylation of this ACP was followed by autoradiography with radiolabeled acetyl CoA and here again only DiSfp showed enzymatic activity (Fig. 7B). HPLC-MALDI-TOF based assays provided further unambiguous analysis. As is evident from figure 8A, DiSfp resulted in a shift of ACP retention time on reverse-phase column from 20.5 minutes to 19.2 minutes. These peaks on MALDI-TOF revealed molecular masses of 10,599 Da and 10,938 Da respectively (Fig. 8B). The increment of 340 Da is consistent with attachment of a phosphopantetheine arm. Similar activity was also observed with hexanoyl and lauroyl CoA, with expected increments in the molecular weight of ACP. ACP modified with hexanoyl CoA showed a retention time of 19.7 minutes and molecular mass of 11036 Da. Lauroyl CoA treated protein eluted at 20.1 minutes and revealed a molecular mass of 11124 Da. As opposed to this, DiAcpS lacked any activity towards this ACP. Intriguingly, DiAcpS failed to modify the type I ACP even with hexanoyl and lauroyl CoA. This is in contrast to our earlier observation with mycobacterial PKSs.

### Role of DiAcpS in Dictyostelium

Our data strongly suggests that multifunctional PKSs are modified by DiSfp. This however raised an issue on the essential requirement of DiAcpS that was reflected in our genetic studies. A BLAST search by using *Saccharomyces* stand-alone ACP with Dictybase identified DDB0184099 as a putative type II ACP, annotated as component of NADPH ubiquinone reductase. The gene was cloned into *E. coli* expression vector and over-expressed protein was purified using affinity chromatography. The protein showed a mobility of ∼10 kDa on SDS-PAGE, less than the expected molecular weight of 13 kDa. The extraction of trypsinized fragments of DiACP on analysis with MALDI-TOF confirmed the identity of protein; however peptides from N-terminus could not be detected (Figure S6). HPLC-purified intact protein analysis with MALDI-TOF showed the molecular weight of this protein as 10109 Da. This deviation from the predicted molecular weight (13195 Da) could be attributed to N-terminus truncation of the protein (approximately 24 amino acids). Interestingly, sequence analysis based on MitoProt software predicted a 0.99 probability of the protein being localized to mitochondria. It is possible that this N-terminus could in fact be a signal for mitochondrial import. A similar scenario has also been observed in *Saccharomyces* ACP where N-terminal leader sequence of about 35 amino acids and is proposed to be toxic for its expression in *E. coli* [35]. *Saccharomyces*, mtACP is known to be involved in octanoate biosynthesis which is a precursor to lipoic acid [36]. MtACP in *Neurospora crassa* has been demonstrated to be essential for the structural integrity of complex I of respiratory chain [37]. It is likely that DiACP may have an analogous role to play in Dicty biology. RT-PCR analysis indeed confirmed presence of DiACP transcript throughout the developmental stages.

Similar to earlier studies with mycobacterial type II ACP, *Dictyostelium* stand-alone ACP (DiACP) also showed activity with DiAcpS and not with DiSfp (Fig. 7C). To unambiguously confirm the phosphopantetheinylation by DiAcpS, modified protein was subjected to HPLC and the eluted protein was analyzed on MALDI-TOF. The protein peak was obtained at a retention time of 19.7 minutes as against 20.7 minutes of *apo*-ACP, and showed an increase in molecular mass from 10109 Da to 10449 Da (Fig. 8C). Similar reaction with hexanoyl CoA also led to a shift in the retention time to 20.4 minutes. As expected, HPLC peak analysis showed an increase of 441 Da in the molecular mass of the protein (Fig. 8D). DiSfp showed no activity with this protein.

Our studies thus demonstrate that DiAcpS and DiSfp exhibit stringent selectivity towards type II and type I ACPs respectively. Previous reports have demonstrated the discriminatory nature of the two classes of PPTases. However, we show here that this specific recognition is embedded in the ACP domain itself rather than being simply dictated by its multi-modular nature. In the case of mycobacterial proteins this specificity was relaxed when longer chain acyl CoAs were used. Whereas no such difference in catalytic function was observed for Dicty ACPs and PPTases strictly adhered to their selectivity criterion even with the other acyl CoAs. This observation underscores the differences in the carrier proteins of the two organisms. Since mycobacterial PKSs are involved in biosynthesis of complex lipids which utilize long-chain lipids, it is possible that mere binding in the active site pocket could initiate this transfer of acyl phosphopantetheine chain. In conclusion, we have shown clear demarcation in the functional roles of two PPTases in *Dictyostelium*. Through their action on PKS/FAS systems, these enzymes are expected to be vital in the initiation and progress of developmental pathway.

## Materials and Methods

### Materials

Genomic DNA was isolated from *D. discoideum* AX2 strain. [1-^14^C] acyl CoAs (55 mCi/mmol) were obtained from American Radiolabeled Chemicals. Non-radioactive acyl-CoA substrates were purchased from Sigma.

### Gene-knockouts and RT-PCR studies

Knockouts were constructed as described by Kuwayama *et al.* [38]. Blasticidin resistance (Bsr) cassette was cloned between the *XbaI-HindIII* sites in pBS from pUC-Bsr vector. Upstream region of the respective PPTases were then cloned as 5′ flanks between *NotI-XbaI* sites. Similarly, downstream regions were cloned as 3′ flanks between *HindIII-KpnI* sites. For constructing *dipks37* knockout cassette, regions around its ACP domain were selected. This construct was then digested with *NotI* and *KpnI* to release the entire knockout cassette (Figure S1). The digested product was column-purified and precipitated. Linear cassette was then transformed into *Dictyostelium* amoeboid cells. Following transformation, cells were aliquoted into 96-wells plates and supplemented with 5 µg/mL blasticidin after 24 hrs. Antibiotic dose was increased to 10 µg/mL after live cells started re-appearing and cells which grew to confluence were analyzed by PCR (Figure S2).

RNA for RT-PCR was extracted from amoeboid cells and developmental stages using TRI reagent and was subsequently treated with DNAse and column-purified using QIAgen kit. RT-PCR was performed using Invitrogen kit with oligodT primer and gene expression checked using gene-specific PCR primers. RT-PCR conditions used were as follows: One cycle of 60 min at 50°C for reverse transcription and one cycle of 30 sec at 98°C, followed by 30 cycles of 15 sec at 98°C, 30 sec at 45°C annealing, and 30 sec at 68°C for extension. The details of the primer sets used are included in Table S1. IG7 (mitochondrial large rRNA) was used as the RT-PCR control.

### Cloning, Expression and Purification of Proteins

The *D. discoideum* PPTases, *diacps* (DDB0217726) and *disfp* (DDB0186752) were amplified from genomic DNA using gene-specific primers and cloned into TOPO cloning vector (Invitrogen). Sequences of all the primers used in this study have been enlisted in Table S1. For expression of N-terminally His_6_-tagged protein, the genes were cloned into pET28(a) (Novagen) and protein expression was checked in BL21(DE3) strain of *Escherichia coli*. For DiAcpS expression, culture was grown at 37°C till O.D. reached 0.6 and was induced with 0.5 mM IPTG at 18°C. DiSfp culture was grown at 37°C till O.D. reached 0.6 and was induced with 0.5 mM IPTG at 30°C. Mycobacterial PKS12 ACP domain of module 1 was PCR amplified from pTC5 [39] and cloned into pBS (Stratagene). The gene was then cloned into pET21(c) (Novagen) and expressed in BL21 cells at 22°C by inducing with 0.5 mM IPTG. DiPKS16 ACP and stand-alone ACP were amplified from *Dictyostelium* genomic DNA and cloned into pBS. Subsequent cloning was done in pET21(c) for expression of C-terminal His_6_-tagged protein. Expression conditions were same as that for the PKS12 ACP. All proteins were purified to homogeneity using Ni^2+^-NTA affinity chromatography.

### HPLC-Mass spectrometry coupled assays

Phosphopantetheinylation assay was set up with 54 µM *apo*-ACP, 34 µM DiAcpS/DiSfp, 12.5 mM MgCl_2_, 250 µM CoA and 50 mM Tris-Cl (pH 8.0), in a final volume of 75 µL [18]. Reaction was quenched with 50 mM EDTA after 60 minutes of incubation at 37°C. 50 µL of this reaction mix was then loaded onto Phenomenex, C18 reverse-phase HPLC column and eluted with 20 minutes linear gradient from 12% ACN to 90% ACN in H_2_O with 0.1% CF_3_CO_2_H. The eluted products were concentrated, resuspended in 50% ACN and 0.1% CF_3_CO_2_H and then analyzed on 4800 MALDI TOF/TOF Analyzer.

### Gel-Binding Assays

Radioactive assays were set up with 57 µM of *apo*-ACP, 20 µM PPTase, 12.5 mM MgCl_2_, 45 µM [1-^14^C] Acetyl-CoA and 50 mM Tris-Cl (pH 8.0), in a final volume of 20 µL. Reaction was quenched directly by adding non-reducing SDS dye after incubation of 20 minutes at 30°C. Samples were loaded on SDS-PAGE, gel was dried and analyzed using a phosphorimager (BAS5001).

### Kinetic Analysis

Kinetic parameters for DiAcpS and DiSfp were determined with 20 µM of either of the PPTase, and 5–45 µM [1-^14^C] acetyl-CoA. Concentration of mycobacterial PKS2 and Rv1344 was fixed at 54 µM. Radiolabeled ACP was quantified using phosphorimager (BAS5001). All the experiments were carried out in triplicates and standard deviations were estimated by using GraphPad.

## Supporting Information

Figure S1 **Generation of** ***diacps*** **,** ***disfp*** **and** ***dipks37*** **knockouts in** ***Dictyostelium*** **by homologous recombination.** Blasticidin resistance cassette (Bsr) was cloned between the XbaI and HindIII sites of pBluescript vector, named as PBS-Bsr vector. Sequences in the 5′ region and 3′ region of *diacps* and *disfp* genes were PCR amplified from genomic DNA and cloned in the NotI/XbaI and HindIII/KpnI of the PBS-Bsr vector, so as to flank the Bsr cassette. *Dipks37* knockout was prepared by cloning the 5′ and 3′ regions of its ACP domain. The vector was digested with NotI and KpnI enzymes to release the knockout cassette. The digested DNA was column-purified, precipitated and transformed into amoeboid cells.
(PDF)

Figure S2 **PCR analysis to checking homologous recombination of knockout cassettes in** ***Dictyostelium*** **amoeboid cells.** A, strategy for confirming homologous recombination. Black shaded blocks represent regions upstream and downstream to the 5′ and 3′ flanks respectively. Numbers above the lanes in panels B, C and D depict the primer numbers used. B, confirmatory PCR for *diacps* knockout. No amplification was seen with primer set 1+2, whereas, expected amplifications were observed for primer sets 1+3 and 2+4. This suggests non-homologous recombination. C, *disfp* knockout clone also shows a similar pattern, indicating non-homologous recombination. D, *dipks37* knockout clone shows expected fragments with all the primer sets, confirming homologous recombination.
(PDF)

Figure S3 **Phenotypic defects observed in** ***dipks37*** **knockout mutants.** *Dipks37* knockout mutant generated by homologous recombination exhibit a similar phenotype as reported by Noel and co-workers. As compared to the wild type slug (A.), mutants (C.) are slender and break apart. Mutant fruiting bodies (D.) also show abnormal phenotype by slopping down on their stalks and giving a messy appearance. Whereas, wild type fruiting bodies (B.) remain erect on their stalks.
(PDF)

Figure S4 **Mass spectrometric identification of DiAcpS and DiSfp –** MALDI-TOF spectra of both DiAcpS and DiSfp is represented along with the list of peptides that were identified.
(PDF)

Figure S5 **Mass spectrometric identification of mycobacterial PKS12 ACP domain and DiPKS16 ACP domain –** MALDI-TOF spectra of both proteins is represented along with the list of peptides that were identified.
(PDF)

Figure S6 **Mass spectrometric identification of Dicty stand-alone ACP (DDB0184099) –** MALDI-TOF spectra of both proteins is represented along with the list of peptides that were identified.
(PDF)

Table S1 **List of primers used.** Gene names are indicated against the primer sequences. FP and RP refer to forward primer and reverse primer respectively.
(PDF)
