# Supplementary material for: Two Functionally Distinctive Phosphopantetheinyl Transferases from Amoeba Dictyostelium discoideum
Source: PLoS One. 2011 Sep 12;6(9):e24262. doi: 10.1371/journal.pone.0024262 (PMC3171403; doi:10.1371/journal.pone.0024262)

Figure S1. Generation of DiAcpS, DiSfp and DiPKS37 knockouts in *Dictyostelium* by homologous recombination

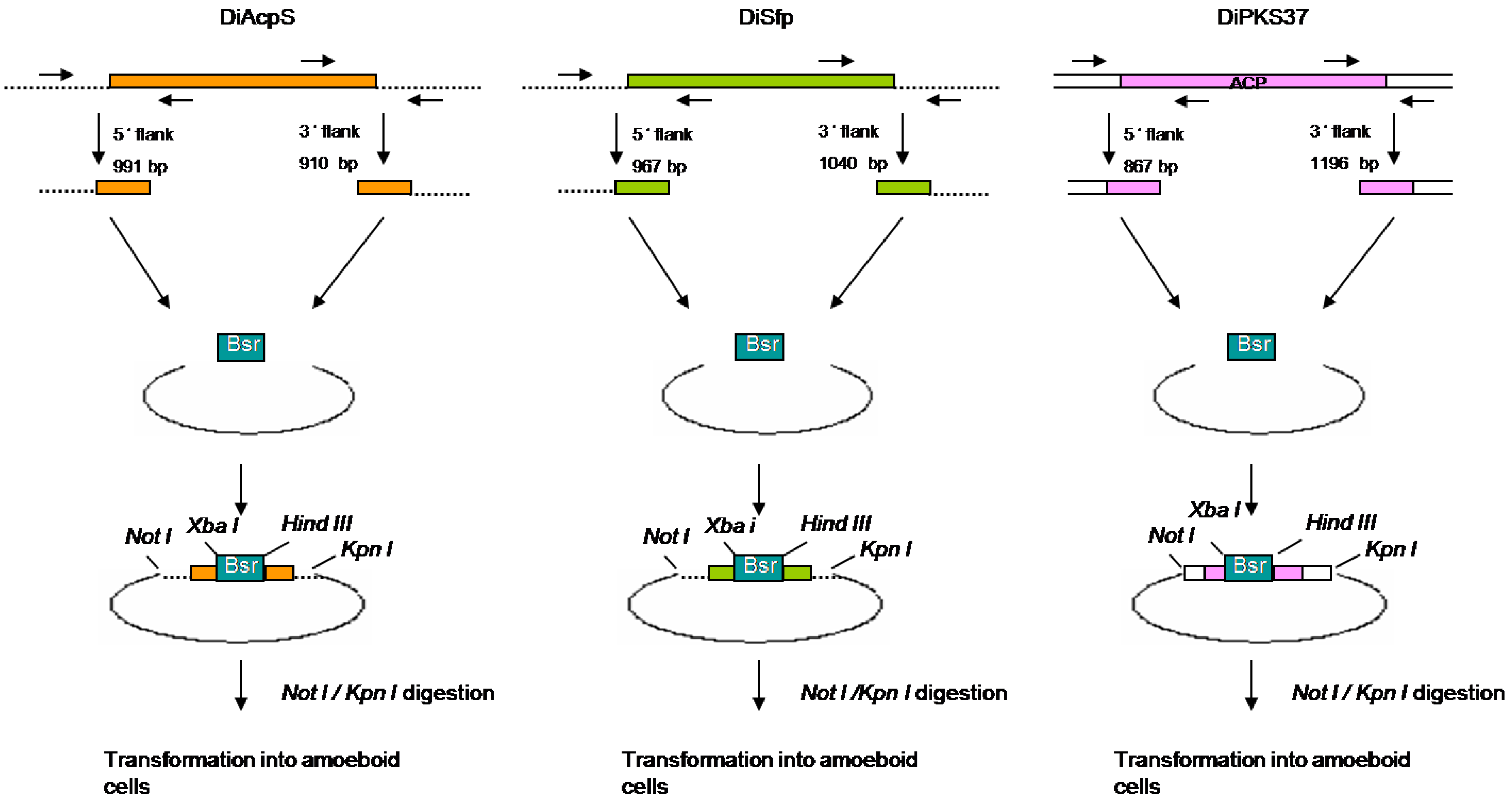

Supplement: Figure S1 — Generation of diacps , disfp and dipks37 knockouts in Dictyostelium by homologous recombination. Blasticidin resistance cassette (Bsr) was cloned between the XbaI and HindIII sites of pBluescript vector, named as PBS-Bsr vector. Sequences in the 5′ region and 3′ region of diacps and disfp genes were PCR amplified from genomic DNA and cloned in the NotI/XbaI and HindIII/KpnI of the PBS-Bsr vector, so as to flank the Bsr cassette. Dipks37 knockout was prepared by cloning the 5′ and 3′ regions of its ACP domain. The vector was digested with NotI and KpnI enzymes to release the knockout cassette. The digested DNA was column-purified, precipitated and transformed into amoeboid cells. (PDF) [file pone.0024262.s001.pdf]
