# Supplementary material for: Two Functionally Distinctive Phosphopantetheinyl Transferases from Amoeba Dictyostelium discoideum
Source: PLoS One. 2011 Sep 12;6(9):e24262. doi: 10.1371/journal.pone.0024262 (PMC3171403; doi:10.1371/journal.pone.0024262)

Figure S2. PCR analysis to checking homologous recombination of knockout cassettes in *Dictyostelium* amoeboid cells

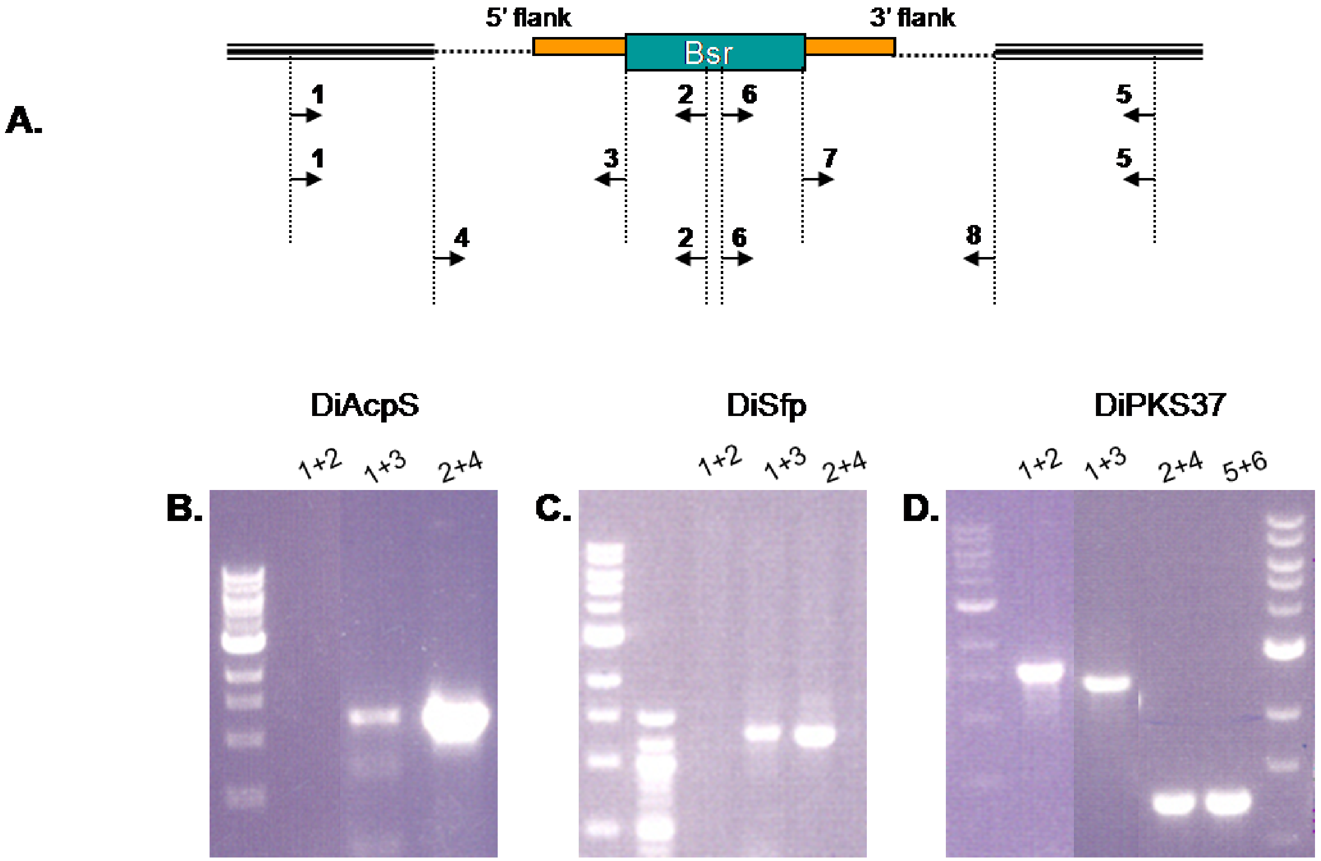

Supplement: Figure S2 — PCR analysis to checking homologous recombination of knockout cassettes in Dictyostelium amoeboid cells. A, strategy for confirming homologous recombination. Black shaded blocks represent regions upstream and downstream to the 5′ and 3′ flanks respectively. Numbers above the lanes in panels B, C and D depict the primer numbers used. B, confirmatory PCR for diacps knockout. No amplification was seen with primer set 1+2, whereas, expected amplifications were observed for primer sets 1+3 and 2+4. This suggests non-homologous recombination. C, disfp knockout clone also shows a similar pattern, indicating non-homologous recombination. D, dipks37 knockout clone shows expected fragments with all the primer sets, confirming homologous recombination. (PDF) [file pone.0024262.s002.pdf]
