# Supplementary figures and images for: Two Functionally Distinctive Phosphopantetheinyl Transferases from Amoeba Dictyostelium discoideum
Source: PLoS One. 2011 Sep 12;6(9):e24262. doi: 10.1371/journal.pone.0024262 (PMC3171403; doi:10.1371/journal.pone.0024262)

Figure S3. Phenotypic defects observed in DiPKS37 knockout mutants

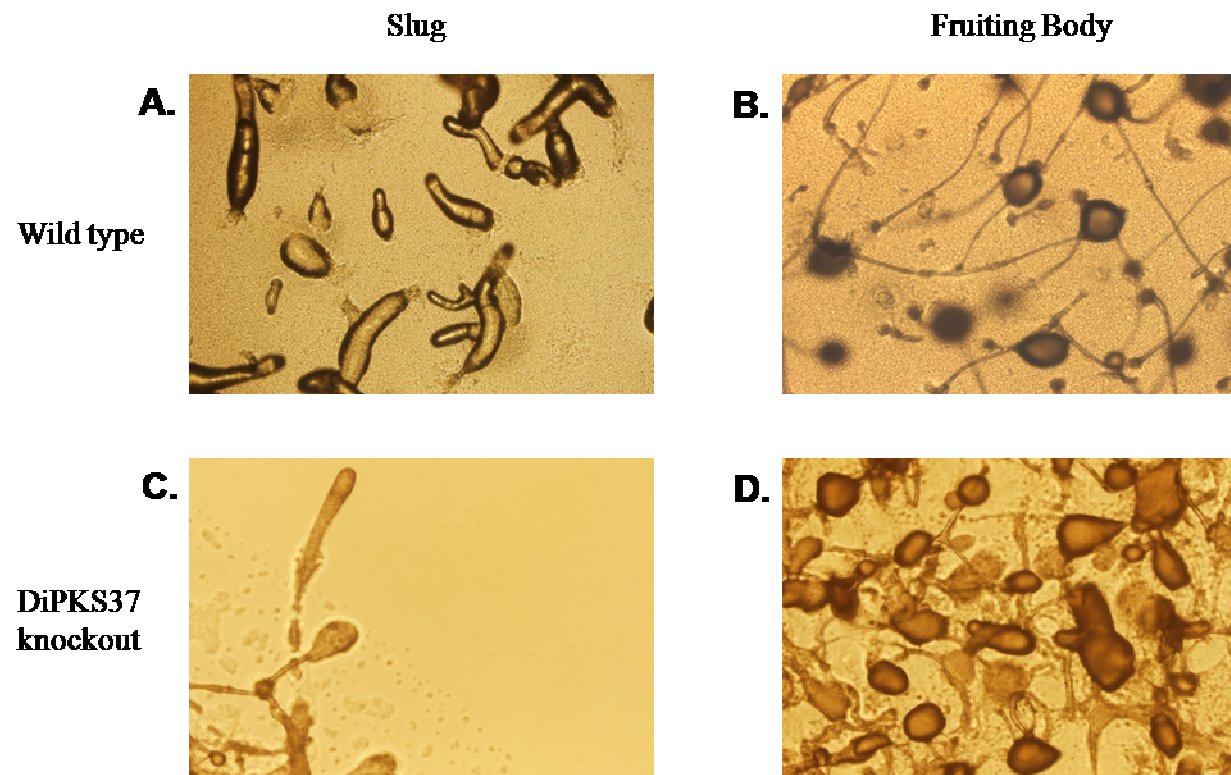

Supplement: Figure S3 — Phenotypic defects observed in dipks37 knockout mutants. Dipks37 knockout mutant generated by homologous recombination exhibit a similar phenotype as reported by Noel and co-workers. As compared to the wild type slug (A.), mutants (C.) are slender and break apart. Mutant fruiting bodies (D.) also show abnormal phenotype by slopping down on their stalks and giving a messy appearance. Whereas, wild type fruiting bodies (B.) remain erect on their stalks. (PDF) [file pone.0024262.s003.pdf]
