# Supplementary material for: Two Functionally Distinctive Phosphopantetheinyl Transferases from Amoeba Dictyostelium discoideum
Source: PLoS One. 2011 Sep 12;6(9):e24262. doi: 10.1371/journal.pone.0024262 (PMC3171403; doi:10.1371/journal.pone.0024262)
Supplement: Figure S4 — Mass spectrometric identification of DiAcpS and DiSfp – MALDI-TOF spectra of both DiAcpS and DiSfp is represented along with the list of peptides that were identified. (PDF) [file pone.0024262.s004.pdf]

Figure S4. MALDI-TOF Identification of DiAcpS and DiSfp

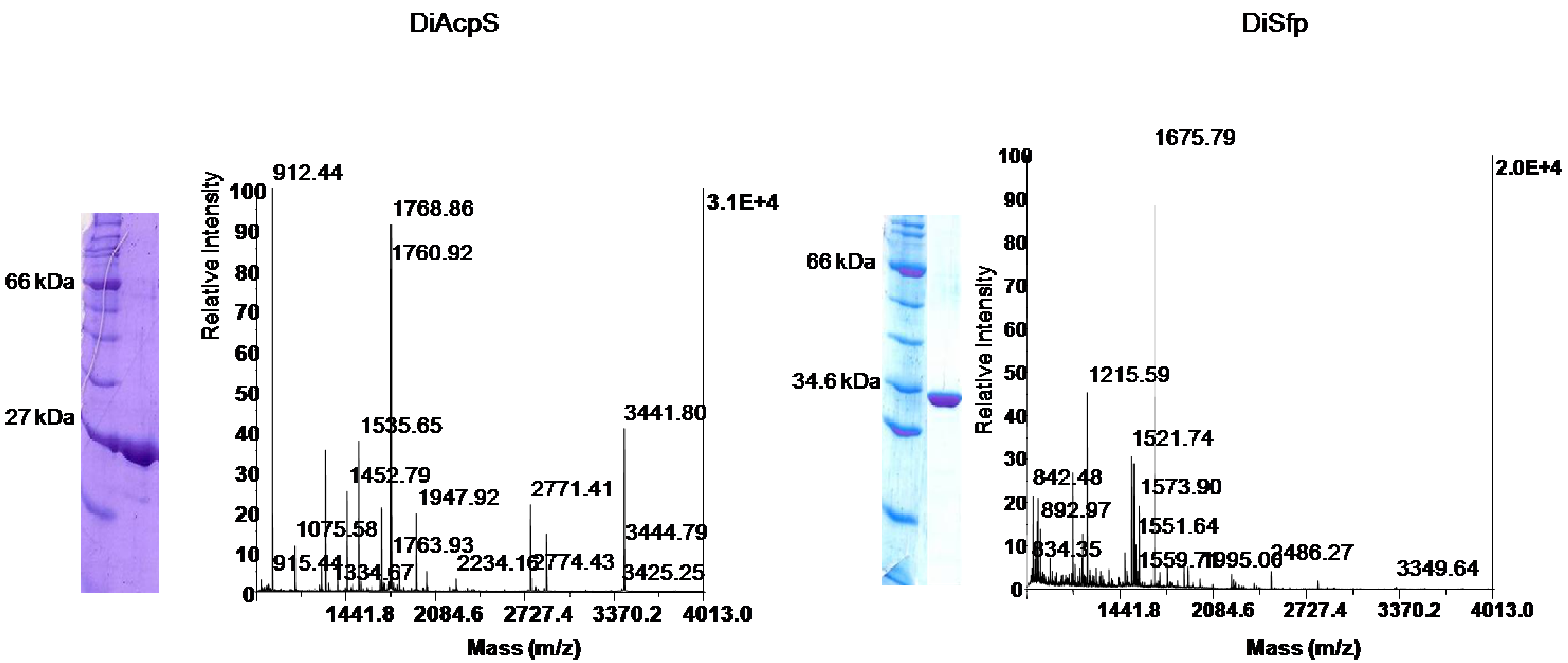

DiAcpS

Observed	Mr(expt)	Sequence
1075.5848	1074.5775	IFGIGNDIVK
1452.7892	1451.7819	RAFNEVEISIFK
1296.6556	1295.6483	AFNEVEISIFK
912.4500	911.4427	GFEYLAGR
2770.4155	2769.4082	IHLAISHDTDYAISNVILESNESTK
2884.4653	2883.4580	IHLAISHDTDYAISNVILESNESTKN

DiSfp

Observed	Mr(expt)	Sequence
1573.8937	1572.8864	ELVNILLLGFDISK
1215.5841	1214.5768	WKPNDIEW
1675.7883	1674.7810	EINDFINDPIESNR
875.4268	874.4195	DGSWLIGK
1115.6377	1114.6304	RLMIELVNK
959.5199	958.5126	LMIELVNK
1183.5736	1182.5663	MLSLDYNSIK
1883.9542	1882.9469	TQSNKPYLSTTTTISNK
2261.8762	2260.8689	MSEFFDTMSSCFTDNEWK
1434.7411	1433.7338	IDLFFIHWCLK
2485.2598	2484.2525	SFEFIIDQTNQTAQIFILNDK
1909.9816	1908.9743	SILNNYQFTYFKPFK
1521.7537	1520.7464	DNLSIQILESDFK
