# Supplementary material for: Two Functionally Distinctive Phosphopantetheinyl Transferases from Amoeba Dictyostelium discoideum
Source: PLoS One. 2011 Sep 12;6(9):e24262. doi: 10.1371/journal.pone.0024262 (PMC3171403; doi:10.1371/journal.pone.0024262)
Supplement: Figure S5 — Mass spectrometric identification of mycobacterial PKS12 ACP domain and DiPKS16 ACP domain – MALDI-TOF spectra of both proteins is represented along with the list of peptides that were identified. (PDF) [file pone.0024262.s005.pdf]

Figure S5. MALDI-TOF identification of PKS12 ACP domain and DiPKS16 ACP domain

PKS12 ACP domain

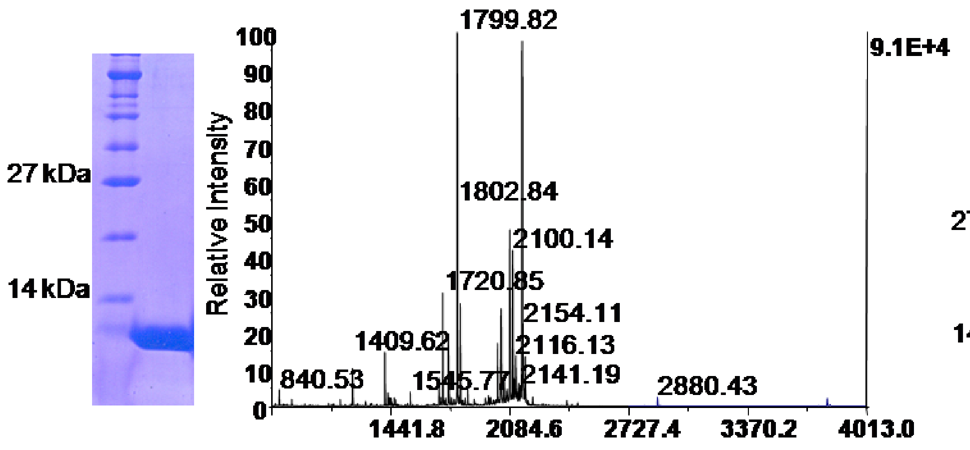

Observed	Mr(expt)	Sequence
1196.5178	1195.5105	DVMSEFPTDR
1460.7300	1459.7227	GLAPDGRCKPYAGR
1003.5594	1002.5521	DLLAPFGDR
1545.7913	1544.7840	NVRQTVLFDQAVR
1235.5996	1234.5923	VAMRAIAANFR
1952.0824	1951.0751	LHGLPEAEQHAVLLGLVR
2017.0714	2016.0641	LHIATVLGNITPEAIDPDK
1799.8573	1798.8500	AFQDLGFDSLTAVEMR
2391.3115	2390.3042	LKSATGLSLSPTLIFDYPTPNR
2150.1560	2149.1487	SATGLSLSPTLIFDYPTPNR
1460.7300	1459.7227	GLAPDGRCKPYAGR
1994.0303	1993.0230	QAVRFADSVRFAQAAGGSR
1133.6196	1132.6123	DGAGVLVVMTR
1331.6971	1330.6898	GEVYTARVHGSR
2156.1479	2155.1406	VHGSRAVGGLLVPPSDRPWR
1842.8265	1841.8192	CAPAALRYLSQARHTGK
1968.3541	1967.3468	HVVARHGVRNLVLVSRR
2061.1111	2060.1038	FDGGTLPPMFVDLINAPTR
1813.8009	1812.7936	AFQELGFDSLTAVEMR

DiPKS16 ACP domain

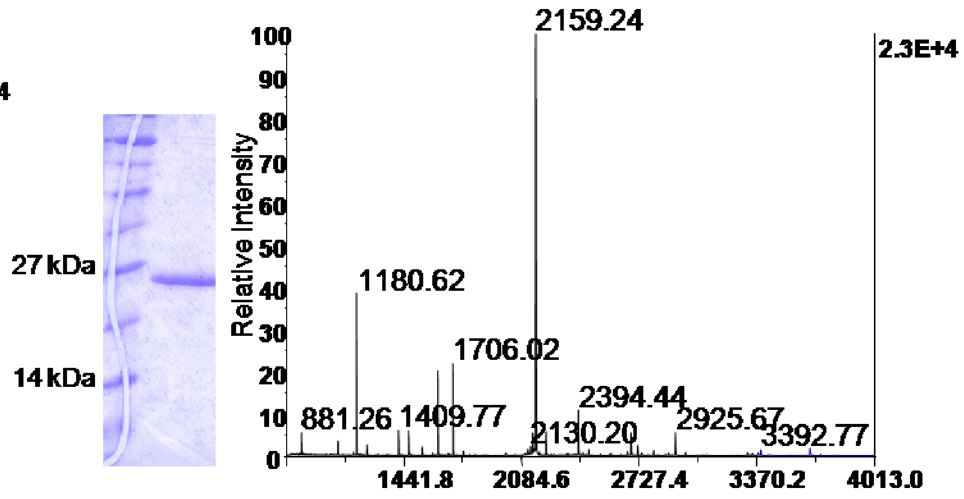

Observed	Mr(expt)	Sequence
2172.2185	2171.2112	SFDAGADGYIRSEGGGVCILK
1681.0050	1679.9977	SNIGHLESAAGIASLIK
1202.6292	1201.6219	DFVKHQTISK
2336.4280	2335.4207	CKPSNSTITSKPIIVSPLHKK
1763.0491	1762.0418	DGVQHHLQTSITPTTK
2260.3413	2259.3340	DSPLTLIENQLIEEKSSESK
2329.2927	2328.2854	TIDSLDQSIDLASLKQVIIEK
1706.0353	1705.0280	LLLPKGQLILMEPPK
3659.8762	3658.8689	DIESMSLTFSSDPESLNSSYSNCIFIVSKEQK
1623.9900	1622.9827	VISTISELLSIHPSK
2336.4280	2335.4207	VISTISELLSIHPSKLNLDTR
1706.0353	1705.0280	LKDYGIDSLLTVQLK
1464.8376	1463.8303	DYGIDSLLTVQLK
1180.6392	1179.6319	NWIDKEFTK
2663.5168	2662.5095	EFTKNLFTHLQLSSSSINSIIQR
2158.2722	2157.2649	NLFTHLQLSSSSINSIIQR
2573.5198	2572.5125	NLFTHLQLSSSSINSIIQRISSK
