# Supplementary material for: Two Functionally Distinctive Phosphopantetheinyl Transferases from Amoeba Dictyostelium discoideum
Source: PLoS One. 2011 Sep 12;6(9):e24262. doi: 10.1371/journal.pone.0024262 (PMC3171403; doi:10.1371/journal.pone.0024262)
Supplement: Figure S6 — Mass spectrometric identification of Dicty stand-alone ACP (DDB0184099) – MALDI-TOF spectra of both proteins is represented along with the list of peptides that were identified. (PDF) [file pone.0024262.s006.pdf]

Figure S6. MALDI-TOF identification of DiACP

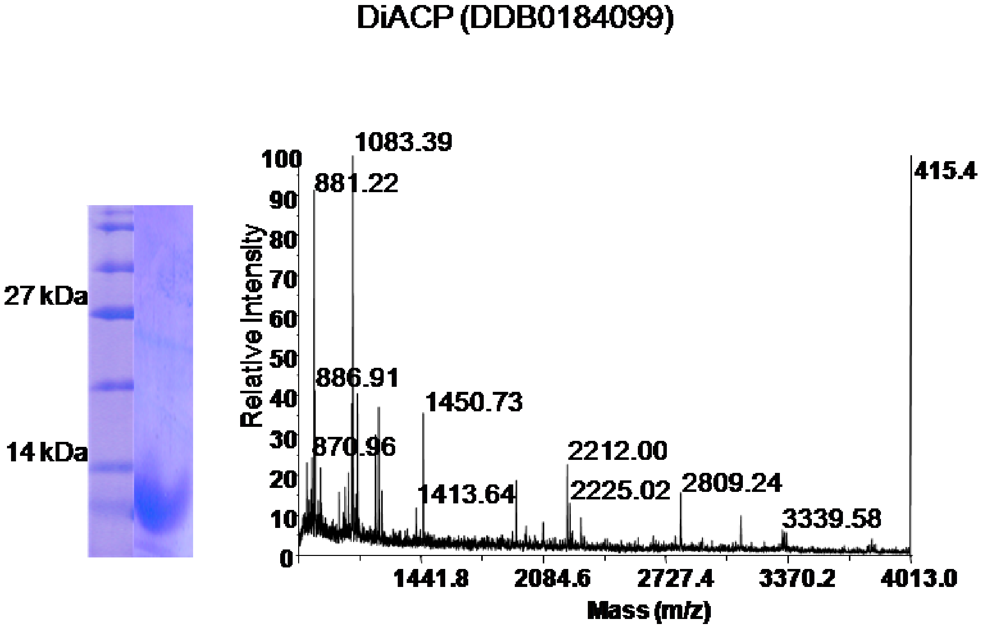

Observed	Mr(expt)	Sequence
1234.5234	1233.5161	QPIVASSMMVR
1107.5453	1106.5380	VIGVVSQYDK
3360.3638	3359.3565	ELGLDSLDSADILVAVEEEFGIEIPDEEADK
1413.6345	1412.6272	ITSCAETISYLR
