# Supplementary material for: Two Functionally Distinctive Phosphopantetheinyl Transferases from Amoeba Dictyostelium discoideum
Source: PLoS One. 2011 Sep 12;6(9):e24262. doi: 10.1371/journal.pone.0024262 (PMC3171403; doi:10.1371/journal.pone.0024262)
Supplement: Table S1 — List of primers used. Gene names are indicated against the primer sequences. FP and RP refer to forward primer and reverse primer respectively. (PDF) [file pone.0024262.s007.pdf]

Table S1

List of primers

DiAcpS Exon1 FP – 5′ AGTGGATCCATGAATAAGATATTTGGTAT 3′
DiAcpS Exon1 RP – 5′ ATTTATCACCATGTCTTTTAAATG 3′

DiAcpS Exon2 FP – 5′ ATCATCATTTAAAAGACATGGTGAT 3′
DiAcpS Exon2 RP – 5′ TAGAAGCTTTTAATTTTTAGTTGATTCAT 3′

DiSfp Exon1 FP – 5′ CACCGGATCCATGAACCAAAATGAAAAAGA 3′
DiSfp Exon1 RP – 5′ CAAAGAATTCACTCATTTTTTGATTACGTGG 3′

DiSfp Exon2 FP – 5′ CACCATGAGTGAATTCTTTGATACAATGTCA 3′
DiSfp Exon2 RP – 5′ ACGAAGCTTTTACTTTATTTTAAAATCTG 3′

DiACP FP – 5′ GGCTAGCATGATTAGAAATACCTTCAAA 3′
DiACP RP – 5′ GCTCGAGTTTGGCAGTTGGTGTTTTTCT 3′

DiPKS16 ACP FP – 5′ CGCCATATGTCATCTGCTAGCTCTGATGATTC 3′
DiPKS16 ACP RP – 5′ TTAAGCTTAAGTTCGGTGCGGATATAGGAGGC 3′

PKS12 ACP FP – 5′ TTCATATGCTGCACGGGCTGCCC 3′
PKS12 ACP RP – 5′ TTAAGCTTAAGTTCGGTGCGGATATAGGAGGC 3′

DiAcpS RT-PCR FP – 5′ ACATGGTGATAAATTTTTAAAAAGAGC 3′
DiAcpS RT-PCR RP – 5′ TTAATTTTTAGTTGATTCATTTGATTCTAA 3′

DiSfp RT-PCR FP – 5′ CACCATGAGTGAATTCTTTGATACAATGTCA 3′
DiSfp RT-PCR RP – 5′ ACGAAGCTTTTACTTTATTTTAAAATCTG 3′
